# State Diabetes Prevention and Control Program Participation in the Health Disparities Collaborative: Evaluating the First 5 Years

**Published:** 2006-12-15

**Authors:** Barbara A Larsen, Maurice “Bud” Martin, David Hutchins, Ana Alfaro-Correa, Laura Shea

**Affiliations:** Utah Heart Disease and Stroke Prevention Program, Utah Department of Health; University of Maine at Farmington, Farmington, Me; Centers for Disease Control and Prevention, Division of Diabetes Translation, Atlanta, Ga; Centers for Disease Control and Prevention, Division of Diabetes Translation, Atlanta, Ga; New York State Department of Health, Diabetes Prevention and Control Program Riverview Center, Albany, NY

## Abstract

**Background:**

Approximately 20.8 million people in the United States, or 7% of the population, have diabetes mellitus. Treatment for this disease costs Americans more than $130 billion yearly, and it is the sixth leading cause of death. The prevalence of diabetes has grown substantially in recent decades and is expected to continue to rise.

**Context:**

The medically underserved and poor are at greater risk of developing diabetes and its complications than are other members of the U.S. population. The Health Resources and Services Administration makes health care resources and services available to economically disadvantaged populations through the Health Disparities Collaborative (HDC), a consortium formed to pool resources and services from state- and community-level donors. Since 1999, many of the Centers for Disease Control and Prevention's Division of Diabetes Translation State Diabetes Prevention and Control Programs (DPCPs) have joined the HDC to leverage resources and services.

**Methods:**

The purpose of a 2004 evaluation was to examine the impact that DPCP involvement with the Collaborative had on aspects of diabetes care at Federally Qualified Health Centers (FQHCs). An electronic survey was administered to DPCP coordinators. They were asked about 1) their roles and experience as participants in the Collaborative; 2) the skills and expertise most useful in developing and maintaining an effective collaboration for improved health care for diabetes; 3) which DPCP contributions were viewed as being routine and which were perceived to be essential; 4) the effects of DPCP contributions on the use of the chronic care model under which FQHCs operate; and 5) which health systems improvements played the greatest role in enhancing components of the chronic care model.

**Consequences:**

Most respondents identified themselves as DPCP coordinators with 3 years of experience in that position. Organizational skills, such as communication, leadership, conflict resolution, negotiation, and meeting management, were cited as necessary to develop and maintain collaborative partnerships. DPCP contributions to FQHCs were perceived to be training, technical assistance with clinical care and patient education, financial resources, linkages to other diabetes partners, educational materials, and improved linkages with community resources.

**Interpretation:**

DPCPs contribute resources, skills, knowledge, and varied perspectives to the Collaborative that FQHCs may not have otherwise.

## Background

Approximately 7% of the U.S. population, a staggering 20.8 million people, has diabetes, which is the sixth leading cause of death in the nation (1). Complications attributable to diabetes mellitus (e.g., blindness, hypertension, heart disease, kidney disease, lower extremity amputations, complications in pregnancy) add to the complexity of the disease and its treatment (1). Treating diabetes costs Americans $92 billion yearly in direct medical costs and another $40 billion yearly in indirect costs such as disability and lost work days (1). The seriousness, complexity, and costs associated with diabetes and its treatment and its disproportionate increase in prevalence among poor, medically underserved, uninsured and underinsured, and high-risk populations necessitate creative yet effective means of prevention, detection, and treatment (2).

## Context

### Introduction: Health Disparities Collaborative

In the 1990s, the Health Resources and Services Administration's (HRSA's) Bureau of Primary Health Care (BPHC) created and began implementing the Health Disparities Collaborative (HDC). The HDC is an innovative, data-driven, public health partnership that has improved care for chronic diseases through improved health care delivery systems among the nation's network of providers that serve the uninsured and underinsured (3). The HDC was formed "to improve access to high quality, culturally and linguistically competent primary and preventive care for underserved, uninsured, and underinsured Americans" (4). The HDC pools health care resources and services at state, local, and community levels in order to deliver them with increased effectiveness and efficiency to FQHCs (3).

Federally Qualified Health Centers (FQHCs) traditionally are the point of contact with providers that deliver direct service to medically underserved and poor patient populations (5). To further maximize resources and services, the chronic care model (CCM) is used to systematically improve the care provided at FQHCs (6).

### Chronic care model

The CCM identifies essential elements of the health care system for providers to focus on and use to organize and encourage high-quality chronic disease care (7). According to the CCM, six components are required to produce interactions between an informed, activated patient and a prepared, proactive multidisciplinary team: 1) organizing patient health care; 2) forming community linkages; 3) encouraging self-management support; 4) maximizing delivery system designs for efficiency; 5) providing patient decision support; and 6) providing improved patient information-sharing systems (4). This model can be applied and implemented in clinical settings for various chronic illnesses and is credited with generating healthier patients, more satisfied providers, and cost savings (7).

With support from HRSA and the BPHC, the Institute for Healthcare Improvement has provided education and training to FQHCs for adoption and use of the CCM. The HDC uses the CCM to focus on several chronic diseases, including cardiovascular disease, depression, asthma, cancer, and diabetes. The HDC has also used the model to address combinations of chronic diseases simultaneously (8).

The diabetes section of the HDC focuses on improving diabetes and pre-diabetes performance measures through improved care delivery systems, increased access, and decreased health disparities among medically underserved populations (8). Of interest for this article is the HDC's use of the CCM as it relates to diabetes treatment and prevention of complications in patients with diabetes who use FQHCs.

### Complementary efforts of CDC and HRSA 

The Division of Diabetes Translation at the Centers for Disease Control and Prevention (CDC) and HRSA share a common goal in treating and preventing diabetes. The agencies have been working as partners through the HDC since 1999. The Division of Diabetes Translation funds 59 Diabetes Prevention and Control Programs (DPCPs) in all 50 states, the District of Columbia, and U.S. territories. These programs are required through cooperative agreements with the Division of Diabetes Translation to address health disparities among people living with diabetes (9). HRSA funds direct services; CDC does not. CDC programs are designed to leverage resources to treat and prevent diabetes and its complications. Both HRSA and CDC share the goals of improving the quality of care and the quality of life of patients with diabetes. The DPCPs are encouraged to join the HDC Diabetes Collaborative (Collaborative) and to share resources with FQHCs to boost collective reach and goal attainment. A conceptual model of this relationship is shown in the Figure.

**Figure:**
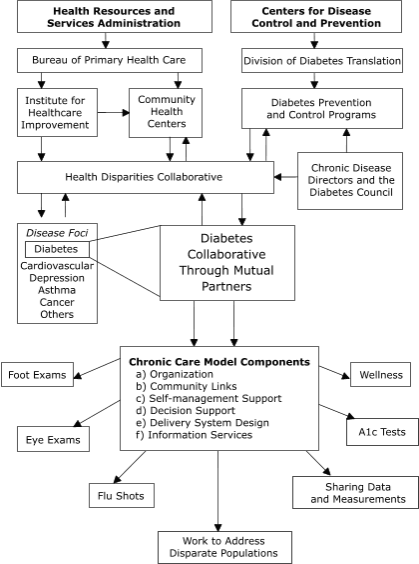
Conceptual model showing how complementary federal agencies can work together to achieve mutual diabetes mellitus prevention and treatment goals through partnerships in the Health Disparities Collaborative.

**Table:** 

### Evaluation

In 2005, the Diabetes Council of the Association of State and Territorial Chronic Disease Program Directors conducted an evaluation to help the organization understand DPCP perceptions about the nature and value of contributions made by the Diabetes Council to FQHCs. They also examined the impact that the Collaborative experience had on aspects of diabetes care as practiced by the FQHCs using the CCM.

## Methods

An evaluation team with expertise in program evaluation or experience with the Collaborative was identified by the Diabetes Council. A participatory approach was used by the team to conceptualize, design, and implement this cross-sectional (data collected at one time), formative evaluation to provide information to guide program improvement. The evaluation was designated as a program evaluation and not as research by the office of the director at CDC and was thus not subject to institutional review. The evaluation was conducted in the winter of 2004 through a short questionnaire sent by e-mail to DPCP coordinators in all CDC-funded states and territories. This initial questionnaire engaged stakeholders and obtained input from the perspective of participants in the Collaborative. A useful and accurate evaluation of Collaborative processes and impacts was developed. The evaluation team developed and distributed a survey instrument by using Survey Monkey (10), a commercial electronic data collection company.

### Instrument 

The survey targeted all 59 DPCP coordinators and consisted of 36 items presented in five sections that queried DPCP coordinators about 1) their roles and experience as participants in the Collaborative; 2) the skills and expertise that were most useful in developing and maintaining an effective collaboration for improved diabetes health care; 3) which DPCP contributions they viewed as routine and which they viewed as essential; 4) the effect of DPCP contributions on components of the CCM used by FQHCs to make health systems improvements; and 5) which health systems improvements enhanced the principal CCM components.

The survey items varied in format (e.g., multiple-choice questions, rating scales with multiple selection matrices, open-ended text responses). Section one explained that participation was voluntary, that the survey would take less than 30 minutes, and that participation would be interpreted as consent to use the data provided in aggregate with other respondents. Respondents were assured of confidentiality, and individual identifiers were required to ensure adequate follow-up.

Section two asked about the respondents' roles with the Collaborative and their number of years of involvement. Sections three and four pertained to the resources, skills, and expertise needed to develop and maintain Collaborative partnerships between DPCPs and FQHCs. Section five dealt with an assessment of DPCP contributions and health systems improvements in relation to the CCM. There were measurements for the benefits and drawbacks of partnering and for indications of needed patient care improvement.

The survey was drafted and pilot-tested on six people who had previous experience with the Collaborative, were no longer actively involved, and would not participate in future data collection. Revisions were made based on feedback from the pilot test.

### Data collection 

The revised survey was forwarded by e-mail to all DPCP coordinators. At each DPCP, the coordinator was instructed to ask the person on staff with the most involvement in the Collaborative to complete and return the survey. Respondents were given 21 days to complete the survey and were sent an e-mail reminder before the closing date. To improve the response rate, the due date was extended approximately 2 weeks.

## Consequences

### Role and experience 

Forty-eight (81%) of the 59 DPCP coordinators surveyed responded to the questionnaire. Eight of the respondents reported that they had not yet participated in the Collaborative and were not included in the analysis. Seventy-five percent of the remaining respondents had participated in the Collaborative for at least 3 years, and 19% had been involved for more than 5 years. When provided a list of options for reporting their primary roles and given the opportunity to "select all that apply," the most frequently selected choices were program liaison or DPCP coordinator (59%), diabetes educational support (54%), and technical assistant (52%). Less commonly identified were quality improvement support (36%) and health systems support (33%).

### Resources and skills 

Respondents were asked to rank DPCP organizational skills (e.g., communication, leadership, conflict resolution, negotiation, meeting management) and technical expertise (e.g., the ability to access community resources and knowledge about quality-improvement methodology, diabetes patient education, data analysis, diabetes clinical care, information management) in terms of their importance for developing and maintaining partnerships with FQHCs. Many organizational skills were perceived as being important, but communications and leadership were ranked as being most useful in developing and maintaining a solid collaboration. The technical expertise area identified as most important to developing and maintaining the Collaborative was the ability to assess community resources. Organizational skills and technical expertise items were identified, and respondents were asked to indicate if a skill or expertise was needed to develop or to maintain Collaborative partnerships. No statistical differences (according to 95% confidence intervals) were shown to indicate different skills were needed to develop versus maintain partnerships (Table 1).

### Essential DPCP contributions 

DPCP respondents were asked to rate the contributions they made to the Collaborative as being routine or essential. Linkages to community resources, educational materials, training, clinical staff exposure to other diabetes partners, technical assistance with clinical care, and technical assistance with patient education were most commonly identified as being essential contributions. These and other DPCP contributions are presented in Table 2.

### CCM components most affected by DPCP contributions 

Respondents were asked whether contributions affected each component of the CCM (e.g., self-management support, decision support, clinical information systems, delivery systems design, organization of health care, links to community resources). They were instructed to select all components affected by each contribution. DPCP coordinators reported self-management support as the CCM component most commonly affected by DPCP contributions.

As shown in Table 3, the greatest contributions to CCM components were in the areas of technical assistance with patient education, educational materials, financial resources, training, and linkages to community resources.

### DPCP impact on health systems improvements 

Respondents were also asked to indicate whether DPCP contributions affected the implementation of health systems improvements for each of the six CCM components. The health systems improvements that played a role in enhancing CCM components are presented in Table 4. Respondents indicated that use of clinical data to monitor indicators was the most common health systems improvement resulting from the DPCP partnership and that data sharing was another common improvement with more than 20% of respondents indicating that data sharing affected each of the six CCM components. Respondents were also asked to indicate which CCM components were supported by each health systems improvement. The CCM component most affected was patient self-management support; more than 15% of respondents checked this column (data not shown).

## Interpretation

In April 2005, the Diabetes Council and the Division of Diabetes Translation evaluated the role and perceptions of DPCP contributions to health systems improvements at FQHCs. The evaluation was based on perceptions of DPCP coordinators and other staff closely involved with Collaborative activities from its inception to the present. Forty-eight representatives from the 59 DPCPs responded to the questionnaire, and the response rate was 81%. Respondents were highly qualified to provide knowledgeable feedback because 75% of those queried were in leadership roles and had been involved with the Collaborative for more than 3 years. Another 19% were involved for more than 5 years. When provided a list of options for reporting their primary roles and given the opportunity to *select all that apply*, most respondents reported that they were the program liaison or DPCP coordinator (59%), diabetes educational support (54%), and technical assistant (52%). Quality improvement support was the primary area for 36% of the respondents, and health systems support was the area reported by 33% of the respondents.

HRSA's and CDC's community partnerships bring to the Collaborative varied resources, multiple perspectives, skills, and expertise. Our findings indicate that DPCPs consider team-building skills (e.g., leadership, meeting management, communication, conflict resolution, negotiation) important for developing Collaborative partnerships between DPCPs and FQHCs. Our findings are analogous to those of Lasker et al (11) and their premise that high-functioning partnerships increase the scope of services offered. These views are supported by the work of Mills and Weeks (12) that showed perceptions of strong team leadership, conflict resolution skills, useful information systems, understanding of other team members, and respect among team members were rated highly by five strong Collaborative project teams.

Respondents indicated that assessing community resources is an important area when working with FQHCs. They also rated quality improvement methodology, patient education, data analysis and reporting, diabetes clinical care, and information management as important areas of expertise needed to develop and maintain partnerships with FQHCs. Quality improvement methodology was cited as a useful collaboration tool, and this factor is consistent with the work of Wilson et al who list "ideas for improvement" and "strategies for learning about and making improvements" (13) as two of seven components from collaborative improvement projects that are important determinants of success.

DPCPs responded that important contributions to FQHCs included providing linkages to community resources and educational materials, training, connecting clinical staff to other diabetes partners, providing technical assistance for clinical care and patient education, and providing financial resources. DPCP coordinators are often trained diabetes educators and are generally knowledgeable about community resources available to support persons with diabetes mellitus. DPCPs typically provide financial support to FQHCs and have access to patient and provider educational materials. They are also well connected to community groups with a stake in diabetes.

Because they must compete for resources, funding streams, and patients, many FQHCs tend to operate with available resources rather than reach out to other community programs, services, and health providers for additional support. Referrals are made internally by using their own staff and clinics. FQHCs are generally understaffed and underfunded, so time constraints may affect their ability to reach out for more resources in their own community.

DPCP respondents perceived patient self-management support that seeks to empower and prepare patients to manage their health and health care to be the CCM component most commonly affected by their contributions to FQHCs. Diabetes control and outcomes depend to a significant degree on the effectiveness of self-management. By using a collaborative approach, providers and patients work together to define problems, set priorities, establish goals, and create treatment plans (6,13).

Although the current CDC approach to reducing the burden of diabetes is not focused on direct services (2), CDC is increasing its impact on health care systems in FQHCs through its partnership with HRSA. FQHCs participating in the Collaborative strive to eliminate disparities and improve delivery of health care by incorporating the CCM into their systems of care. DPCPs play an important role by improving linkages between FQHCs and community resources. Participation in the Collaborative allows DPCPs to affect the care provided to high-risk communities by leveraging resources that influence the FQHCs' health system.

The ways DPCPs reported using resources to support the work of FQHCs varied widely. Some DPCPs dedicated most of their time and resources to self-management education with less effort on other CCM components. Others chose to expend energy largely on linkages to community resources. This response variability is testimony to the unique situation each FQHC faces.

We recognize that, because each Collaborative situation is unique, it may not be possible to generalize the findings reported here since they may not be appropriate for other circumstances. As additional DPCPs and other state-based programs (e.g., those for asthma, cardiovascular health, cancer) begin to work with FQHCs, our findings may informally identify and direct new initiatives. Training to improve expertise in clinical care, patient education, quality improvement methodology, and information management are areas that have been, and likely will be, supported through partnerships with the Division of Diabetes Translation. Providing guidance and coaching to DPCPs to strengthen skills in team building, partnering, and data collection are expected to continue to be useful. The CDC Division of Diabetes Translation might help improve support for DPCP participation in the Collaborative by strengthening its relationship with HRSA through improved data sharing.

HRSA and CDC are complementary agencies with shared goals and have come together through the diabetes section of the HDC to improve diabetes mellitus treatment and prevention. This collaboration illustrates the impact that DPCP contributions can have. By using the CCM as a guide, DPCPs can create an effective bridge between agencies through which the partners can accomplish the business of diabetes treatment and prevention. Involvement in these collaborative efforts has helped FQHCs position themselves to reach HRSA and CDC goals by 1) improving their capacity and ability to reach medically underserved and economically disadvantaged populations; 2) increasing the number of patients receiving hemoglobin A1c tests; 3) reducing hemoglobin A1c concentrations at participating clinics; and 4) increasing data collection and data sharing with DPCPs (14,15).

## Figures and Tables

**Table 1:** Perceptions of DPCP Respondents About Skills and Expertise Needed to Develop and Maintain Collaborative Partnerships, Diabetes Health Disparities Collaborative Evaluation, 2004

Skills and Expertise	% Respondents Perceiving Skills as Important for Developing the Collaborative (95% CI) (n = 36)	% Respondents Perceiving Skills as Important for Maintaining the Collaborative (95% CI) (n = 37)
**Organizational skills**		
Communication (active listening)	100 (100-100)	100 (100-100)
Leadership	100 (100-100)	97 (91-102)
Conflict resolution	87 (78-99)	90 (79-99)
Negotiation	94 (86-101)	92 (83-100)
Meeting management	91 (81-100)	89 (79-99)
**Technical expertise**		
Accessing community resources	94 (87-102)	97 (91-102)
Quality-improvement methodology	89 (77-98)	89 (79-99)
Patient education	86 (74-97)	89 (79-99)
Data analysis and reporting	80 (66-93)	87 (76-97)
Diabetes clinical care	80 (66-93)	84 (72-95)
Information management	77 (63-90)	78 (64-91)

DPCP indicates Diabetes Prevention and Control Program; CI, confidence interval.

**Table 2:** Perceptions of DPCP Respondents (n = 34) About Contributions of the Diabetes Health Disparities Collaborative, Diabetes Health Disparities Collaborative Evaluation, 2004

Contributions	% Respondents Perceiving Contributions as Routine^a^	% Respondents Perceiving Contributions as Essential
Linkages to community resources	18	53
Training	12	50
TA clinical care	21	44
TA patient education	26	41
TA quality improvement	24	24
TA information technology	9	26
Financial resources	18	35
Data collection, analysis and reports	9	24
Literature reviews	9	9
Computers or software	12	24
Exposure of clinic staff to other diabetes partners	29	47
Educational materials	35	53

DPCP indicates Diabetes Prevention and Control Program; CI, confidence intervals; TA, technical assistance.

b Routine contributions were defined as *other than essential* and included minor, random, and nonessential areas.

**Table 3:** Perceptions of DPCP Respondents (n = 34) About DPCP Contributions to Components of the Chronic Care Model, Diabetes Health Disparities Collaborative Evaluation, 2004

DPCP Contributions to Chronic Care Model Components	% Respondents Perceiving DPCP Activities as Contributions to Components of the Chronic Care Model					

Patient Self-management Support	Patient Decision Support	Clinical Information Systems	Delivery Systems Design	Organization of Health Care	Links to Community Resources
Linkages to community resources	76	12	28	34	34	90
Training	79	71	54	71	64	61
TA clinical care	68	68	39	43	36	36
TA patient education	100	48	31	41	28	69
TA quality improvement	48	63	48	70	59	33
TA information technology	30	37	78	48	44	15
Financial resources	81	56	67	48	52	59
Collect data, analysis, and reports	35	50	77	42	50	15
Literature reviews	33	52	22	33	22	22
Computers and software	33	41	74	33	30	26
Exposure of clinical staff to partners	64	57	36	46	46	93
Educational materials	100	61	29	50	46	71
Mean %	62	51	49	47	42	49

DPCP indicates Diabetes Prevention and Control Program; TA, technical assistance.

**Table 4:** Perceptions of DPCP Respondents (n = 34) About the Role of Health Systems Improvements on Components of the Chronic Care Model (CCM), Diabetes Health Disparities Collaborative Evaluation, 2004

Health Systems Improvements	% Respondents Perceiving Health Systems Improvements asContributions to Components of the CCM					

Patient Self-management Support^a^	Patient Decision Support	Clinical Information Systems	Delivery System Design	Organization of Health Care	Links to Community Resources
Patient reminders	52	11	30	30	11	7
Provider reminders	15	48	30	37	15	11
Patient self-management education	93	23	17	17	17	57
Patient referrals to community resources	57	7	3	13	10	83
Use of peer educators	52	17	3	28	17	34
Use of clinical data to monitor indicators^b^	37	70	67	27	30	20
Data sharing^b^	38	48	59	31	34	24

a More than 20% of respondents noted this component was enhanced by each of the health systems improvements.

b More than 20% of respondents noted this health systems improvement supported all six CCM components.

## References

[1] (2005). Centers for Disease Control and Prevention. National diabetes fact sheet: general information and national estimates on diabetes in the United States, 2005.

[2] Murphy D, Chapel T, Clark C (2004). Moving diabetes care from science to practice: the evolution of the National Diabetes Prevention and Control Program. Ann Intern Med.

[3] Health Disparities Collaboratives: home.

[4] Our mission.

[5] Centers for Medicare and Medicaid Services.

[6] Models for changing practice.

[7] Wagner EH (1998). Chronic disease management: what will it take to improve care for chronic illness?. Eff Clin Pract.

[8] Overview.

[9] State-based diabetes prevention and control programs.

[10] (1999). SurveyMonkey.com.

[11] Lasker RD, Weiss ES, Miller R (2001). Partnership synergy: a practical framework for studying and strengthening the collaborative advantage. Milbank Q.

[12] Mills PD, Weeks WB (2004). Characteristics of successful quality improvement teams: lessons from five collaborative projects in the VHA. Jt Comm J Qual Saf.

[13] Wilson T, Berwick DM, Cleary PD (2003). What do collaborative improvement projects do? Experience from seven countries. Jt Comm J Qual Saf.

[14] Kaytura F-A Reducing disparities in diabetes care among the nation's most vulnerable populations. Conference proceeding from the NACHC Annual Convention and Community Health Center Institute.

[15] Mukhtar Q, Jack L, Martin M, Murphy D, Rivera M (2006). Evaluating progress toward Healthy People 2010 national diabetes objectives. Prev Chronic Dis.

